# Melatonin modulates neuroinflammatory response and microglial activation in mice exposed to dim blue light at night

**DOI:** 10.3389/fphar.2024.1416350

**Published:** 2024-05-30

**Authors:** Chao Song, Zhaotaize Suo, Zixu Wang, Jing Cao, Yulan Dong, Yaoxing Chen

**Affiliations:** ^1^ College of Veterinary Medicine, China Agricultural University, Beijing, China; ^2^ The High School Affiliated to Renmin University of China, Beijing, China

**Keywords:** d-BL, melatonin, MT2, microglia, neuroinflammation

## Abstract

**Objectives:**

Dim light at night contributes to neurodegenerative diseases by causing neuroinflammation. In the central nervous system, the activation of microglia is a significant contributor to neuroinflammation. Therefore, there is an urgent need to find an intervention to treat the neuroinflammatory response caused by dim light at night. Melatonin is a rhythmic hormone whose synthesis is suppressed during the day. In this study, we attempt to explore whether and how melatonin improves hippocampal neuroinflammation in mice exposed to dim blue light at night.

**Materials and Methods:**

*In vivo*, a total of 36 male C57BL6/J mice that exposed to no light at night, dim blue light at night, and dim blue light at night with melatonin treatment. *In vitro*, the corticosterone-induced BV2 cells with or without melatonin treatment were used.

**Results:**

Both *in vivo* and *in vitro* experiments showed melatonin treatment significantly reduced dim blue light -induced hippocampal microglial activation and the expression of inflammatory factors IL-1β and TNF-α. This improved effect of melatonin is related to its receptor MT2 rather than MT1. The MT2 blockers significantly increased mRNA levels of M1-type activation marker CD86 and inflammatory cytokines IL-1β and TNF-α in melatonin-treated BV2 cells. Binding of melatonin to its receptor MT2 downregulated the expression of inflammatory proteins P-P65 and NLRP3, consequently inhibited the CD80 expression and M1-type activation in microglia. Furthermore, consistent with the decrease in microglial activation and inflammatory response after melatonin treatment, we also observed a reduction in hippocampal neuron loss and damage to the HT22 cells.

**Conclusion:**

Our findings suggested that melatonin may regulate microglial polarization through MT2/NF-kB-NLRP3 pathway and improves dim blue light -induced hippocampal neuroinflammation in mice.

## 1 Introduction

The term “blue light” refers to the type of light emitted by mobile phones, computers, and other electronic devices, which falls within a potent spectrum on the light spectrum (Zhao et al., 2018; Calvo-Sanz and Tapia-Ayuga, 2020). Despite its dim appearance in dark environments or during nighttime use, screen light can profoundly affect human health and wellbeing (Silvani et al., 2022). As artificial lighting becomes increasingly prevalent, extensive research has delved into its health implications, especially focusing on the neurophysiological effects of dim blue light at night (dBL). Liu et al. (2022) have highlighted the capacity of dBL to induce spatial memory impairment in mice, an effect mediated by neuroinflammation and oxidative stress within the hippocampus, underscoring the vulnerability of this brain region to light-induced perturbations. The significance of these findings is echoed by González (2018) and Fonken et al. (2012; 2013b), who have documented the broad physiological and cognitive disruptions caused by dBL, including exacerbated inflammatory responses and adverse metabolic effects, thus providing a compelling narrative on the health risks associated with night-time exposure to artificial lighting.

In this context, melatonin is highlighted as a compound with significant potential for therapeutic applications due to its potent antioxidative, anti-inflammatory, and neuroprotective properties. Studies by Xu et al. (2020) and Ali et al. (2020) illustrate melatonin’s efficacy in modulating immune responses, particularly in attenuating neuroinflammation by regulating microglial activity, a mechanism critical for mitigating the adverse effects of environmental stressors like dBL. These investigations align with broader research efforts, such as those by Zheng et al. (2021) and Wang et al. (2021), which further elucidate melatonin’s role in downregulating inflammatory pathways and protecting against cognitive deficits.

The intricate interplay between melatonin and microglial activation (Hardeland R, 2021; Guo er al., 2023). Through an integrative analysis, we explore the neuroprotective effects of melatonin against dBL-induced hippocampal inflammation and microglial activation, drawing upon the foundational work of Gao et al. (2023), which underscore the hormone’s capacity to modulate neuroinflammatory responses. Moreover, the study by Wongprayoon and Govitrapong (2015) highlights melatonin’s ability to counteract neuroinflammation through receptor-mediated pathways, providing a molecular basis for its therapeutic potential. Our investigation provides a comprehensive understanding of how melatonin interacts with the neuroinflammatory cascade triggered by dBL and elucidates the underlying mechanisms of its protective effects.

## 2 Materials and methods

### 2.1 Treatment of laboratory animals

In order to replicate a typical daytime environment, a total of 36 male C57BL6/J mice (Vitonglihua, Beijing) were exposed to white LEDs (150 lux) during the day. The mice were exposed to low levels of blue light (5 lux, peak wavelength: 444 nm) all night long throughout the night phase. After the 10th week of exposure to evening light, a 4-week intraperitoneal injection of melatonin (20 mg/kg body weight with melatonin dissolved in anhydrous ethanol) was given. After a 1-week adaptation period, the mice were randomly divided into three groups: (1) LD group: *n* = 12; a light (∼150 lux)/dark (0 lux) cycle, (2) dBL group: *n* = 12; a light (∼150 lux)/dim blue light (∼5 lux) cycle, (3) dBL + Melatonin group: *n* = 12; a light (∼150 lux)/dim blue light (∼5 lux) cycle, melatonin: 20 mg/kg body weight. The experiment was conducted according to the Guide for the Care and Use of Experimental Animals published by the Animal Welfare Committee of the Agricultural Research Organization of China Agricultural University (Approval number: AW92303202-2-2).

### 2.2 Immunohistochemical staining

Iba1 antibody that was resistant to rabbits was incubated overnight on paraffin slices of the mouse hippocampus (1:500, ab178846, Abcam). On the second day, the sections were cleaned with PBS and incubated for 2 hours with biotinylated goat anti-rabbit immunoglobulin G (IgG, 1:100, A0277, Beyotime, Shanghai, China). Following washing, the tissue would be processed for 1.5 h at room temperature using streptavidin-horseradish peroxidase (1:300, A0303. Beyotime in Shanghai, China). Finally, DAB was used to observe the color development. A termination reaction was triggered by a positive signal. The slide was then sealed and the nucleus stained with hematoxylin. Utilizing a microscope (BX51, Olympus, Tokyo, Japan), the location and distribution of immunoreactive chemicals inside the hippocampal region were observed.

### 2.3 Real-time reverse transcription-polymerase chain reaction (RT-PCR)

The TRIzol reagent (CW0580; Vazyme, Nanjing, China) was used to extract total mRNA from liver tissues and HepG2. RevertAid First Strand cDNA Synthesis Kit (R312-01; Vazyme, Nanjing, China) was used to perform reverse transcription on the total mRNA in order to produce cDNA. AceQ qPCR SYBR Green premix (Q111-02, Vazyme, Nanjing, China) was used to perform RT-PCR amplification with chosen gene primers. The relative mRNA level of each sample was standardized to the expression level of the reference gene Gapdh after each sample was examined in triplicate. Table 1 contains a list of the RT-PCR primers.

**TABLE 1:** Primers used for real-time PCR analysis and expected product length.

Gene	Accession no.	Primer sequence (5′to 3′)		Length (bp)
*mIl-1β*	NM_008361.4	F: TGC​CAC​CTT​TTG​ACA​GTG​ATG	R: TGA​TGT​GCT​GCT​GCG​AGA​TT	138
*mTnf-a*	NM_013693.3	F: CCA​CGT​CGT​AGC​AAA​CCA​C	R: TTG​AGA​TCC​ATG​CCG​TTG​GC	90
*mIl-4*	NM_021283.2	F: CTC​GAA​TGT​ACC​AGG​AGC​CAT	R: ACT​CTC​TGT​GGT​GTT​CTT​CGT​T	153
*mIl-10*	NM_009716.3	F: GCA​TGG​CCC​AGA​AAT​CAA​GG	R: ACA​CCT​TGG​TCT​TGG​AGC​TTA​TTA	226
*mCd86*	NM_019388.3	F: ATG​GAC​CCC​AGA​TGC​ACC​AT	R: CCA​GCT​CAC​TCA​GGC​TTA​TGT	260
*mCd206*	NM_008625.2	F: TCA​GCT​ATT​GGA​CGC​GAG​G	R: TGA​GAA​TCT​GAC​ACC​CAG​CG	144
*mCxcl1*	NM_008176.3	F: ACT​GCA​CCC​AAA​CCG​AAG​TC	R: TGG​GGA​CAC​CTT​TTA​GCA​TCT​T	114
*mCxcl2*	NM_009140.2	F: TGCCGGCTCCTCAGTGCT	R: GCC​TTG​CCT​TTG​TTC​AGT​ATC​TTT​TG	281
*mGapdh*	NM_001289726.2	F: ACT​CAG​GAG​AGT​GTT​TCC​TCG	R: TGA​AGG​GGT​CGT​TGA​TGG​C	140

F = forward primer; R = reverse primer.

### 2.4 Western blot assay

RIPA lysate (CW2333S, CWBIO) with 1% phosphatase inhibitor (CW2383S, CWBIO) and 1% protease inhibitor (CW2200S, CWBIO) was used to lyse the liver tissues and BV2 cells. The lysate was centrifuged at 12,000 g for 15 min at 4°C. Using a protein assay kit (CW0014, CWBIO), the supernatant’s protein concentration was measured. Subsequently, the proteins were loaded onto an 8%–12% gradient polyacrylamide gel, electrophoretically transferred to a PVDF membrane (Millipore, Billerica, MA, United States), and primed for incubation with primary antibodies (P-P65, 1:1,000, TP56372, Abmart; CD80, 1:1,000, 66406-1-Ig, Proteintech; NLRP3, 1:1,000, TD7438, Abmart; β-actin, 1:8,000, 66009-1-Ig, Proteintech). The membranes were rinsed with TBST and treated for 1.5 h with horseradish peroxidase-conjugated goat anti-rabbit IgG (1:8,000; CW0103, COWIN) or goat anti-mouse IgG (1:8,000; CW0102, COWIN). The embossed bands were scanned and examined, and the IOD was calculated using ImageJ (Scion Corp., Frederick, MD, United States). The relative protein level in the Ctrl group *in vivo* or in the control cells *in vitro* was defined as 100%. The protein level was adjusted to the density ratio of β-actin.

### 2.5 Double labeling immunofluorescence

Primary antibody against CD80 (1:500, 66406-1-IG, Proteintech) was incubated overnight at 4°C on paraffin slices of the hippocampus and BV2 cell slides. After rinsing the slices in PBS, they were incubated for 2 hours at room temperature with anti-mouse Alexa Fluor 488 (1:300, P0188, Beyotime). After 30 min of room temperature incubation with 5% goat serum, sections were left overnight at 4°C to be exposed to rabbit Iba1 primary antibody (1:1,000, ab178846, Abcam). After that, the slices were cleaned in PBS and left to incubate for 2 hours at room temperature with the anti-rabbit Alexa Fluor 594 (1:300, P0179, Beyotime). Finally, DAPI (1 μg/mL, C0065, Solarbio, Beijing, China) is used to stain the nuclei for 15 min. Utilizing a microscope (BX51, Olympus, Tokyo, Japan), the distribution and position of immunoreactive materials in microglia were examined.

### 2.6 Cell culture and treatment

The BV2 cells were grown at 37°C with 5% CO2 in high-glucose DMEM supplemented with 10% fetal bovine serum (FBS) and 1% penicillin/streptomycin. Prior to receiving medication, the cells were cultured for 6 hours in complete culture medium (DMEM) containing 10% FBS after being injected into either six- or 96-well plates. Following that, the cells were cultured for 12 h in DMEM (base medium) without FBS. The MT1-selective melatonin receptor antagonist Luzindole (HY-101254; MCE, China), the MT2-selective melatonin receptor antagonist 4P-PDOT (HY-100609; MCE, China), and the corticosterone (HY-B1618; MCE, China) were all individually dissolved in DMSO. Melatonin (M5250; Sigma, United States) should be dissolved in anhydrous ethanol. The cells were treated with corticosterone (5 μM) for 12 h, then pretreated with Luzindole (1 μM) and 4P-PDOT (1 μM) for 30 min, respectively, and then treated with Melatonin (200 nM) for 12 h. The protein content was evaluated on a 6-well plate. HT22 cell activity was measured with CCK8. First, for a whole day, BV2 cells were treated to 200 nM melatonin and 5 μM corticosterone. After centrifuging the cells for 10 min at 1,000 rpm to eliminate cell precipitation, HT22 cells were treated with the culture media that was left over. After being injected into 96-well plates, the HT22 cells were grown for 6 hours in complete media and 12 hours in basic medium. The HT22 cells were then given a 24-h treatment with BV2 media. Lastly, the CCK8 assay was used to evaluate cell viability.

### 2.7 Statistical analysis

SPSS version 25.0 (IBM Corp., Armonk, NY, United States) was used to analyze the data, and the results were presented as means ± standard errors. To statistically analyze the group differences, One-way ANOVA was employed. A criterion of *p* < 0.05 was established for statistical significance.

## 3 Results

### 3.1 Melatonin can reduce hippocampal neuroinflammation in mice exposed to dim blue light at night

**FIGURE 1:**
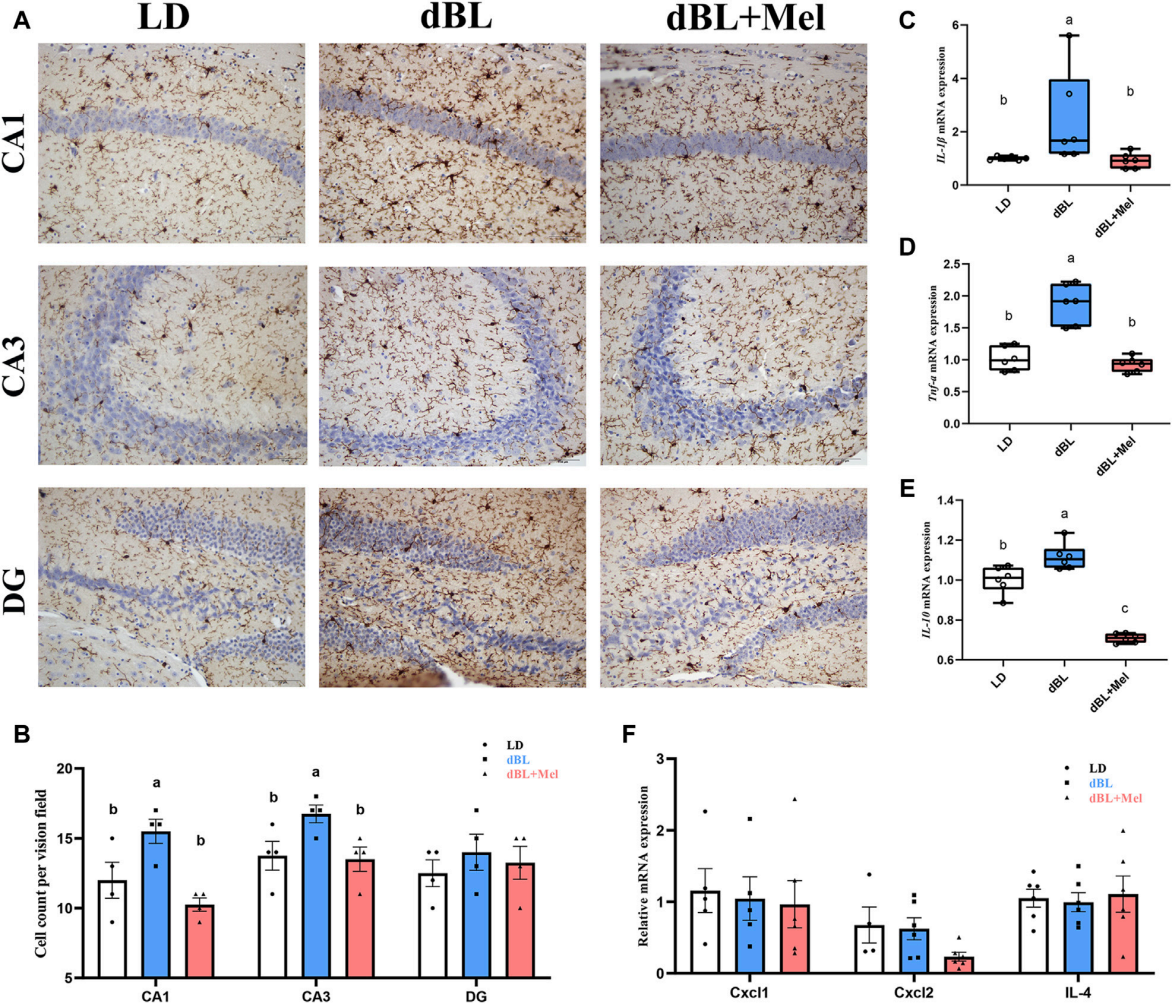
Melatonin can reduce hippocampal neuroinflammation in mice exposed to dim blue light at night. **(A)** Immunohistochemistry of mouse hippocampal microglia in different groups. **(B)** The number of microglia in CA1, CA3 and DG regions of mouse hippocampus. **(C–F)** The mRNA levels of IL-1β, TNF-α, Il-10, Cxcl1, Cxcl2, and Il-4 (*n* = 6). The outcome is the mean ± standard error of the mean. Values (a,b,c and d) without a common superscript letter differ significantly at *p* < 0.05, but values (a,b and c) with the same letter do not differ significantly at *p* ≥ 0.05. LD, dark at night; dBL, dim blue light at night; Mel, Melatonin.

First, to examine the potential impact of melatonin on hippocampal neuroinflammation induced by dim light at night, we quantified the number of CA1, CA3, and DG microglia in the hippocampus of mice across various groups (LD, dBL, and dBL + Melatonin groups). The results showed that the number of microglia in CA1 and CA3 regions of hippocampus in melatonin group was significantly reduced by 23.87% (*p* = 0.002) and 19.40% (*p* = 0.023), respectively, compared with dBL group (Figure 1B). The occurrence of inflammation is often accompanied by the release of inflammatory factors. Therefore, we detected changes in the mRNA levels of IL-1β, TNF-α, IL-4, and IL-10 in the hippocampus of mice. The results showed that the mRNA expression levels of IL-1β, TNFα, and IL-10 were significantly increased by 423% (*p* = 0.041), 118% (*p* = 0.001), and 39.3% (*p* = 0.008), respectively, compared with the LD group (Figures 1C–E). After melatonin treatment, the increase in inflammatory factors induced by blue light was significantly inhibited. Specifically, IL-1β, TNFα, and IL-10 were reduced by 75.7% (*p* = 0.044), 50.9% (*p* < 0.001), and 40.7% (*p* < 0.001), respectively (Figures 1C–E). The mRNA levels of Cxcl1, Cxcl2, and IL4 did not show significant differences between the groups (Figure 1F).

### 3.2 Melatonin improved the loss of hippocampal neurons in mice exposed to dim blue light

**FIGURE 2:**
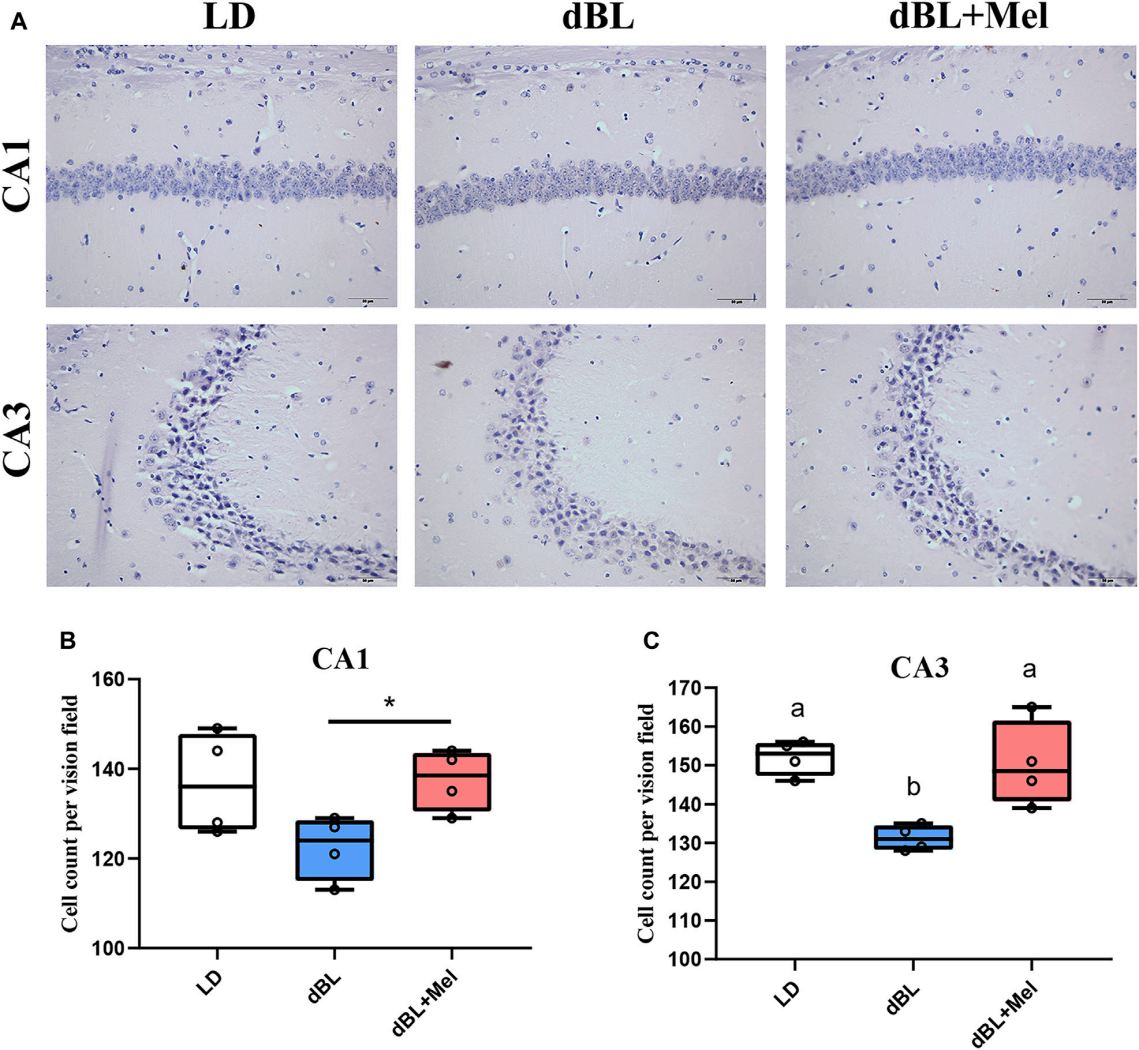
Melatonin improved the loss of hippocampal neurons in mice exposed to dim blue light. **(A)** Nith staining of mouse hippocampus. **(B)** Neuron density in the CA1 region of the mouse hippocampus. **(C)** Neuron density in the CA3 region of the mouse hippocampus. The outcome is the mean ± standard error of the mean. Values (a,b,c and d) without a common superscript letter differ significantly at *p* < 0.05, but values (a,b and c) with the same letter do not differ significantly at *p* ≥ 0.05. LD, dark at night; dBL, dim blue light at night; Mel, Melatonin.

The hippocampus is a crucial structural component for learning and memory functions in mice, and the decline in these functions is frequently linked to neuronal loss. Overactivation of microglia impairs the morphology and function of neurons. Next, we examined the effects of melatonin on hippocampal neuron density in mice. Mouse Nissl staining is shown in Figure 2A. Compared with the LD group, the density of neurons in the hippocampal CA3 region decreased by 10.60% (*p* = 0.001) in the dBL group. The loss of hippocampal neurons in the dBL group may be the result of overactivation of microglia. The neuronal density in the CA1 and CA3 regions of the hippocampus in the melatonin treatment group was significantly increased by 11.57% (*p* = 0.023) and 13.53% (*p* = 0.016), respectively, compared with the dBL group.

### 3.3 Melatonin regulates hippocampal inflammation by inhibiting the activation of microglia

**FIGURE 3:**
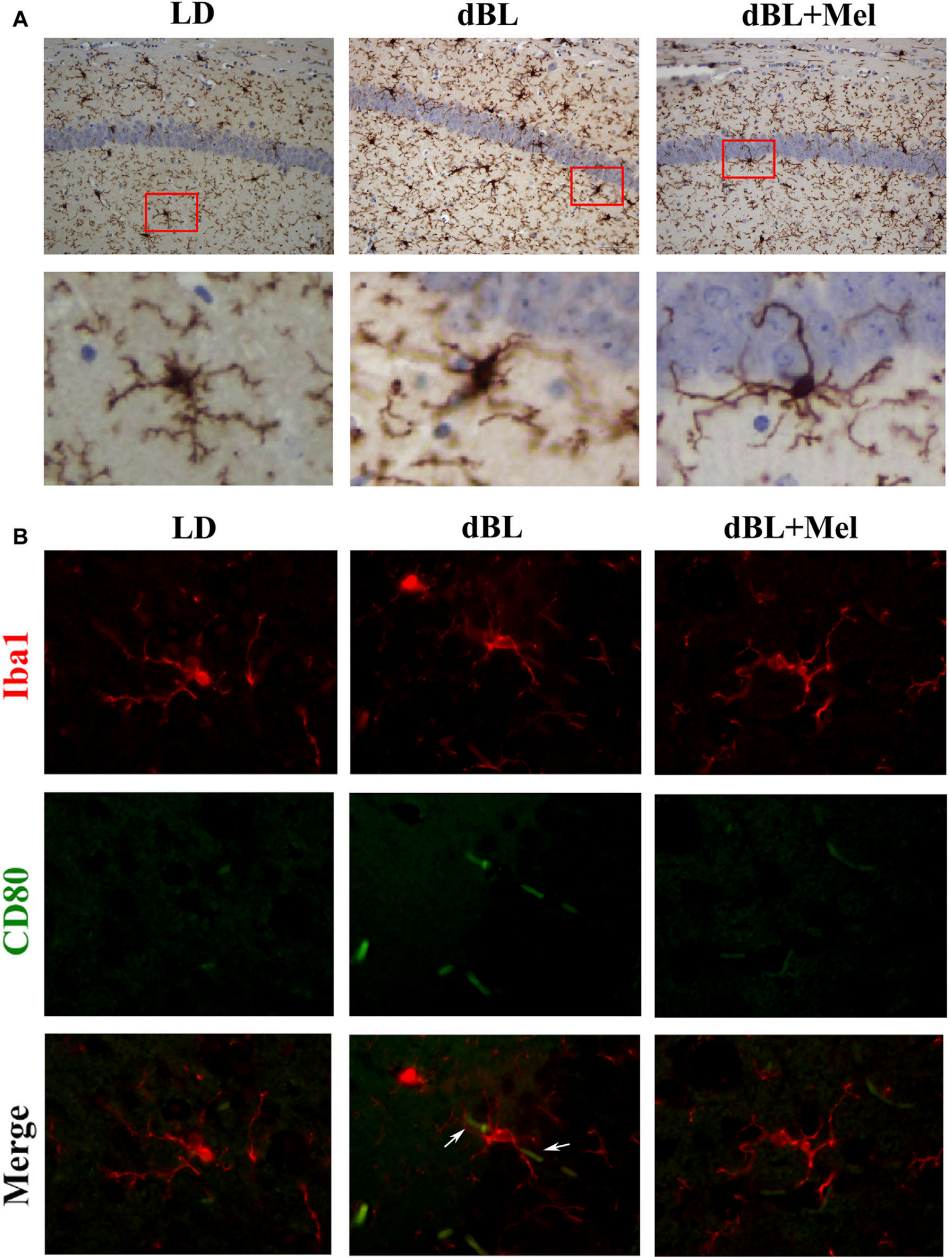
Melatonin regulates hippocampal inflammation by inhibiting the activation of microglia. **(A)** Immunohistochemical diagram of microglia in different groups. **(B–G)** Fluorescence colocalization of CD80 and Iba1 in the mouse hippocampus. Red fluorescent label Iba1 (594 nm) and green fluorescent label CD80 (488 nm). LD, dark at night; dBL, dim blue light at night; Mel, Melatonin.

As the primary line of immune defense in the central nervous system, microglia play a crucial role in the inflammatory response. The activation of microglia *in vivo* is accompanied by significant morphological changes. Our staining results revealed that the cell bodies of hippocampal microglia in the dBL group were enlarged, and the number of branches was significantly reduced. This suggests that exposure to dark blue light at night significantly enhances the activation of hippocampal microglia. However, melatonin supplementation significantly mitigated the blue light-induced morphological changes in microglia (Figure 3A). In addition, we demonstrated the expression of the M1-type marker CD80 on hippocampal microglia using dual immunofluorescence labeling. Immunofluorescence results showed that the expression of CD80 in the microglia of the mouse hippocampus was significantly increased in the blue light group (Figure 3B). Melatonin supplementation reversed the expression of CD80 on microglia, suggesting that melatonin could effectively inhibit the activation of microglia caused by blue light, thereby reducing the level of inflammation in the hippocampus of mice.

### 3.4 Melatonin inhibits the M1-type activation of BV2 by inhibiting the activation of NF-kB and NLRP3

**FIGURE 4:**
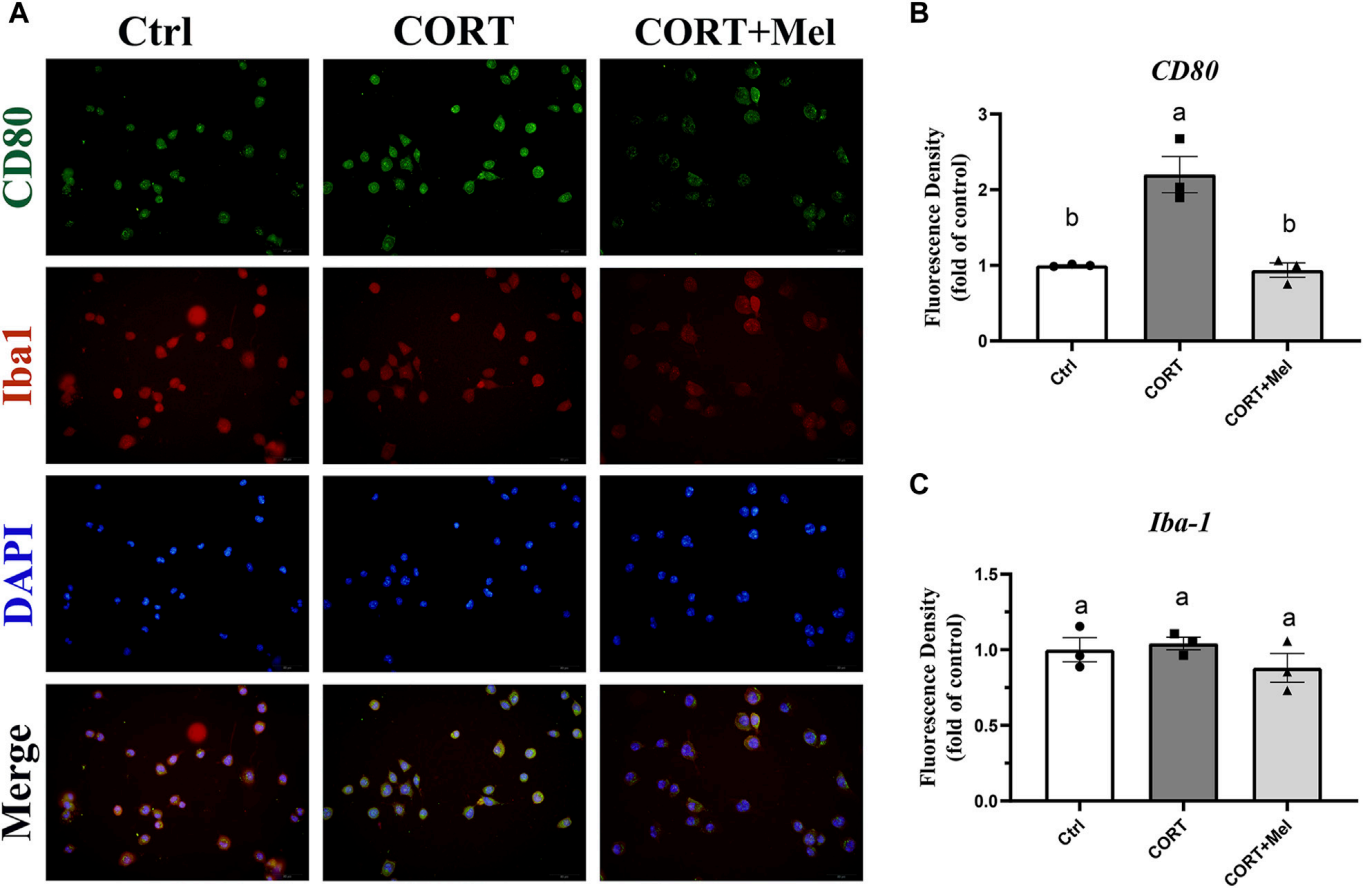
Melatonin inhibits the M1-type activation of BV2 cells induced by corticosterone. **(A)** Immunofluorescence localization of Iba1 and CD80 in BV2 cells, red fluorescence labeled Iba1 (594 nm), green fluorescence labeled CD80 (488 nm), and DAPI labeled nucleus. **(B,C)** Fluorescence intensity analysis of CD80 and Iba1 (*n* = 3). The outcome is the mean ± standard error of the mean. Values (a,b,c and d) without a common superscript letter differ significantly at *p* < 0.05, but values (a,b and c) with the same letter do not differ significantly at *p* ≥ 0.05. CORT, corticosterone; Mel, Melatonin.

**FIGURE 5:**
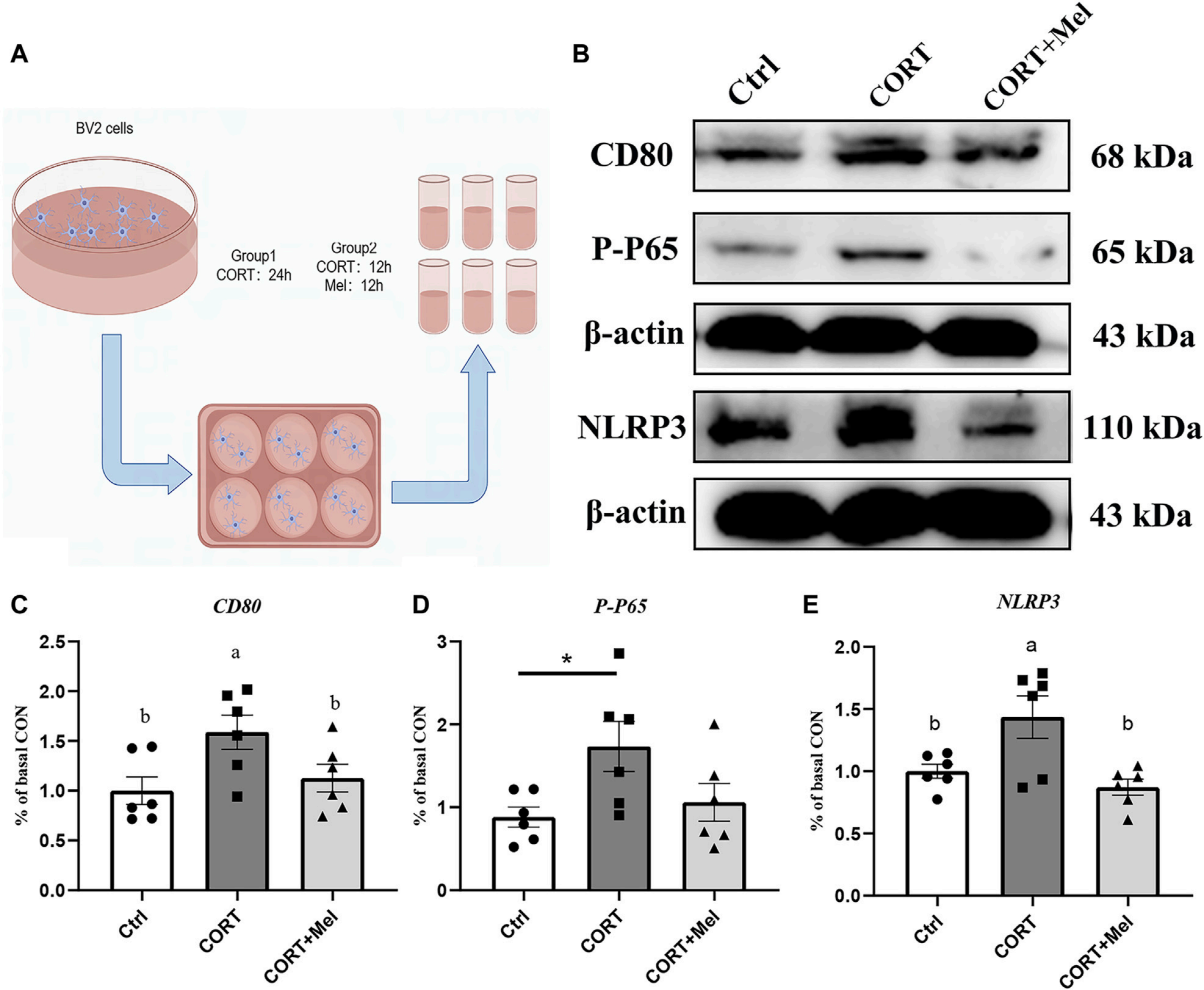
Melatonin reduces M1-type activation of BV2 by inhibiting the activation of NF-kB and NLRP3. **(A)** Schematic diagram of *in vitro* test. **(B–E)** Relative protein levels of CD80, P-P65, and NLRP3 in hippocampus (*n* = 6). The outcome is the mean ± standard error of the mean. Values (a,b,c and d) without a common superscript letter differ significantly at *p* < 0.05, but values (a,b and c) with the same letter do not differ significantly at *p* ≥ 0.05. CORT, corticosterone; Mel, Melatonin.

After determining the regulatory effect of melatonin on microglia in mice, we continued our investigation of melatonin’s role using the BV2 cell line. We simulated the effects of blue light on microglia by stimulating BV2 cells with corticosterone. Our previous results showed that exposure to dim blue light at night significantly increased plasma corticosterone levels and activated hippocampal microglia (Liu et al., 2022). BV2 cells were labeled with Iba1, and then the expression of CD80 was quantified after various treatments (Figure 4A). The results showed that corticosterone and melatonin treatment had no significant effect on the fluorescence intensity of Iba1 in BV2 cells (Figure 4C). However, the fluorescence intensity of CD80 was significantly enhanced after corticosterone treatment, indicating that corticosterone promoted the activation of BV2 cells to the M1 type (Figure 4B). After melatonin supplementation, the expression of CD80 was significantly reduced. This suggests that melatonin may regulate corticosterone-induced BV2 cell inflammation by decreasing the M1-type activation of microglia. We treated BV2 cells with corticosterone and melatonin, as shown in Figure 5A, and then detected the protein expression of CD80, p-P65, and NLRP3. The results showed that the expression of CD80 (56%, *p* = 0.018), PP65 (96.6%, *p* = 0.026), and NLRP3 (43.6%, *p* = 0.036) in BV2 cells significantly increased after corticosterone stimulation (Figures 5B–E). Melatonin can significantly inhibit the activation of NF-kB/NLRP3. The protein levels of p-p65 and NLRP3 in BV2 cells supplemented with melatonin are significantly downregulated compared to the corticosterone group (Figures 5B–E).

### 3.5 The viability of HT22 cells was significantly improved by adding BV2 culture medium exposed to melatonin

**FIGURE 6:**
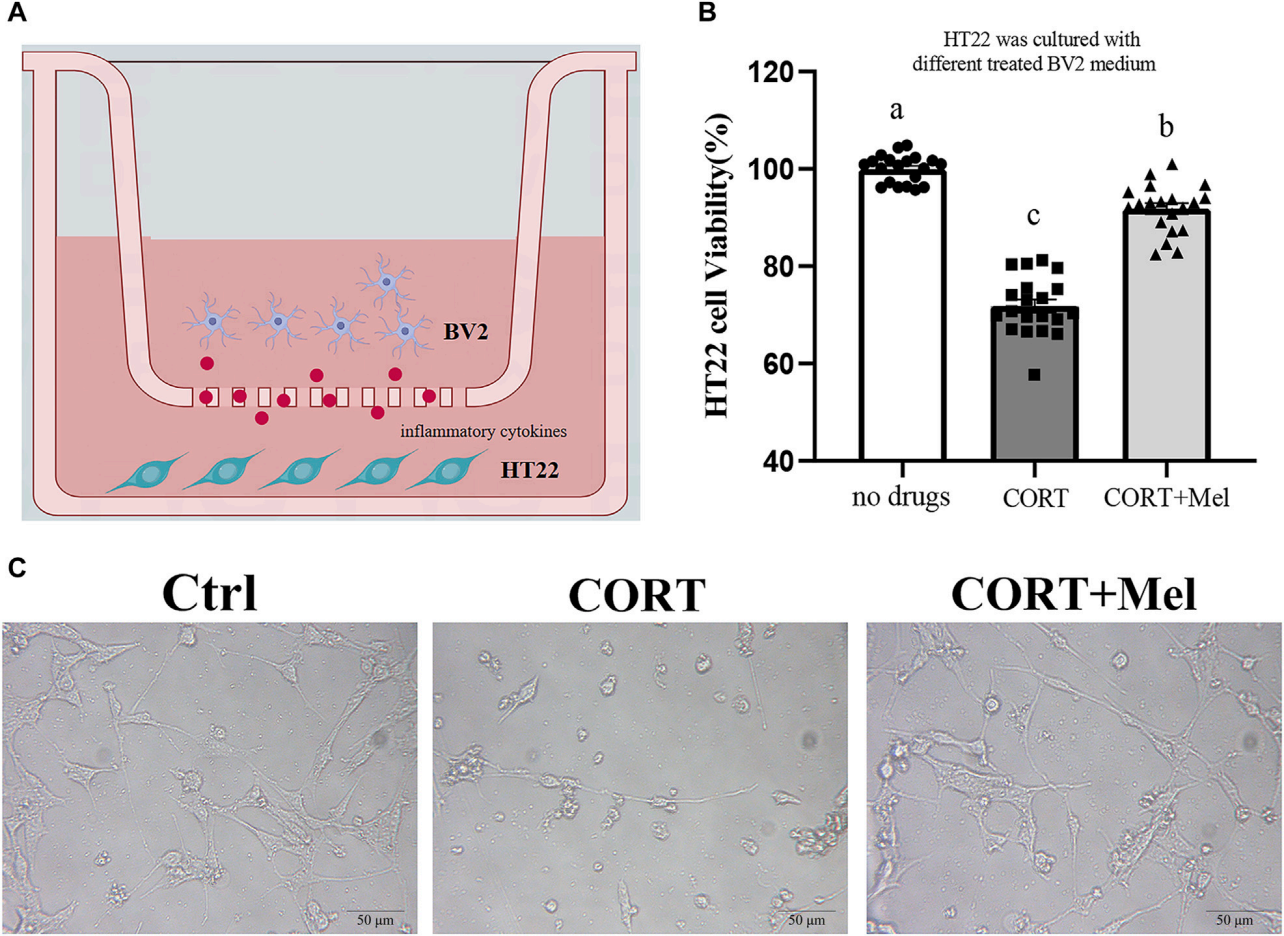
The viability of HT22 cells was significantly improved by adding BV2 culture medium exposed to melatonin. **(A)** Schematic diagram of *in vitro* test. **(B)** The CCK8 method was used to detect the activity of HT22 cells exposed to different BV2 medium (*n* = 20). **(C)** Photomicrograph of HT22 cells treated with different BV2 medium. The outcome is the mean ± standard error of the mean. Values (a,b,c and d) without a common superscript letter differ significantly at *p* < 0.05, but values (a,b and c) with the same letter do not differ significantly at *p* ≥ 0.05. CORT, corticosterone; Mel, Melatonin.

The interaction between microglia and neurons plays a key role in maintaining brain health. Therefore, we proceeded to culture HT22 cells in BV2’s medium to investigate the various effects of microglia on neurons (Figure 6A). First, the results of the CCK-8 assay showed that the cell activity of HT22 was significantly inhibited (29.16%, *p* < 0.001) in BV2 culture containing corticosterone (Figure 6B). In addition, the number of HT22 cells attached to the wall was significantly reduced in the corticosterone group (Figure 6C). The activity of HT22 cells in the melatonin treatment group significantly increased by 30.27% (*p* < 0.001) compared to the cells in the CORT group (Figure 6B).

#### 3.6 Melatonin inhibits the activation of BV2 through MT2 and reduces the occurrence of inflammation

**FIGURE 7:**
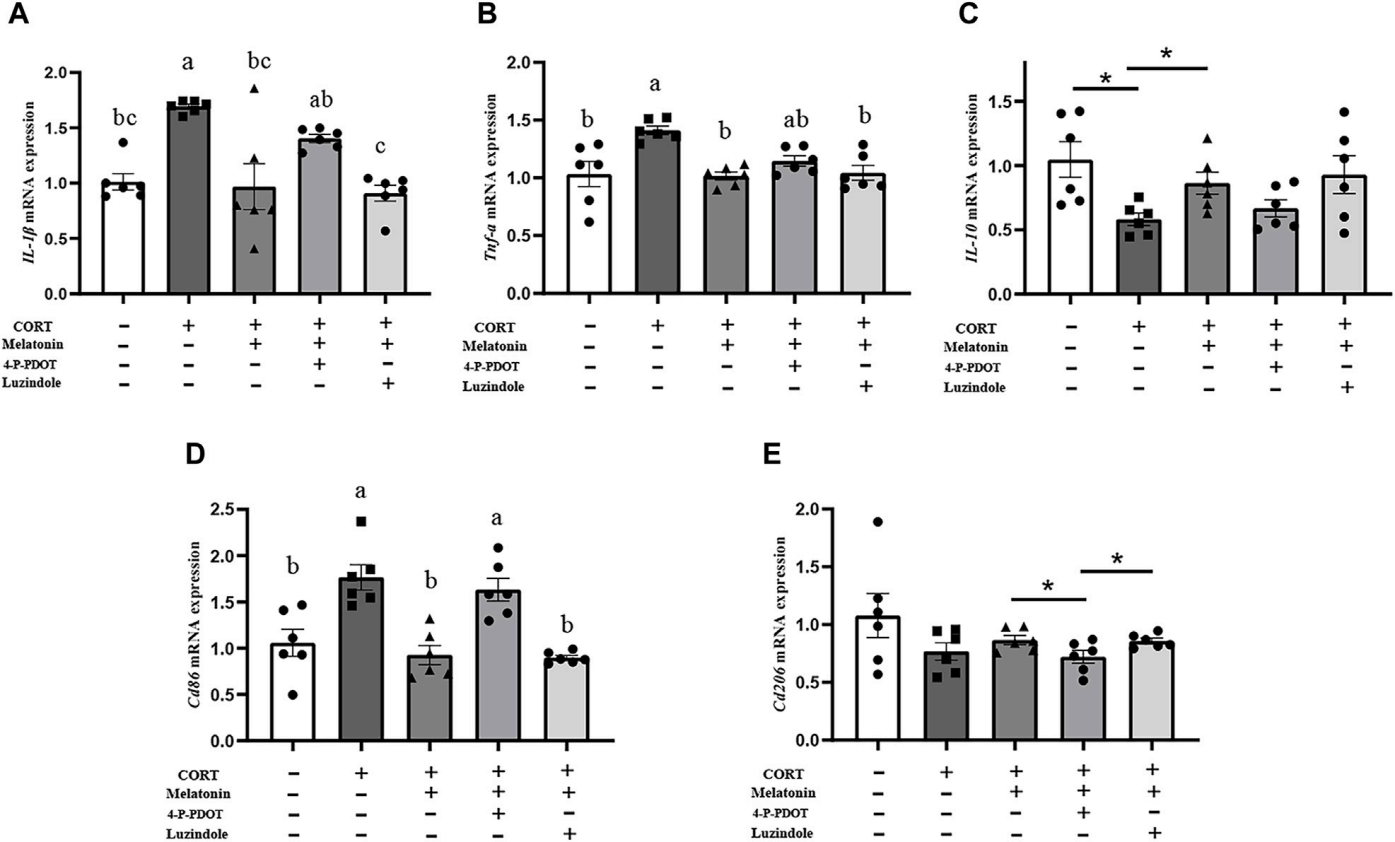
Melatonin inhibits the activation of BV2 through MT2 and reduces the occurrence of inflammation. **(A–E)** The mRNA levels of IL-1β, TNF-α, IL-10, Cd86, and Cd206 in BV2 cells treated with CORT, Meltonin, Luzindole, and 4-P-PDOT (*n* = 6). The outcome is the mean ± standard error of the mean. Values (a,b,c and d) without a common superscript letter differ significantly at *p* < 0.05, but values (a,b and c) with the same letter do not differ significantly at *p* ≥ 0.05. CORT, corticosterone, Mel, Melatonin; Luzindole, MT1 receptor blockers; 4-P-PDOT, MT2 receptor blockers.

To explore the pathway of melatonin action, we added MT1 and MT2 receptor blockers, Luzindole and 4-P-PDOT, to BV2 cells, respectively. First, mRNA levels of IL-1β, TNF-α, and CD86 in BV2 cells were significantly reduced, and mRNA expression of IL-10 was significantly increased after melatonin treatment compared with the corticosterone-stimulated group (IL-1β, 44.25%, *p* = 0.006; TNF-α, 27.54%, *p* < 0.001; Cd86, 47.56%, *p* = 0.001; IL-10, 36.05%, *p* = 0.018; Figures 7A–D). The mRNA levels of IL-1β and Cd86 significantly increased after 4-P-PDOT was added to BV2 cells compared to the melatonin treatment group (IL-1β, 64.95%, *p* = 0.013; Cd86, 76.16%, *p* = 0.001). When luzindole was added to BV2 cells, there was no significant effect on the action of melatonin. This suggests that melatonin can influence the activation of microglia to M1 type through MT2 receptors.

## 4 Discussion

Nighttime exposure to artificial lighting (Dim Light at Night) and shift work have been shown to disrupt normal physiological activities in the brain and body (González, 2018). The study discovered that dLAN of 5 lux influences neuronal dendritic shape in the CA1 and DG areas of the hippocampus, influencing learning and memory function (Fonken et al., 2012). Recent studies have shown that dim light at night can trigger an inflammatory response in the central nervous system (Chen et al., 2021; Liu et al., 2022). Microglia are a type of glial cell, which constitutes the central nervous system’s most crucial immune defense line. Microglia perform essential functions, such as environmental perception and protection against internal and external stimuli. Thus, microglia play an important role in maintaining brain homeostasis and synaptic function, depending on the balance between the M1 and M2 phenotypes of microglia (Rasia-Filho et al., 2023). Overactivation of microglia can lead to dysregulation of synaptic plasticity and neurotoxicity. Age and sex factors also appear to influence the effects of nighttime dim light on the neuroinflammation. Chen et al.’s study showed that adolescent mice exposed to nighttime light exhibited higher levels of pro-inflammatory factors in the hippocampus. Female mice were found to be more susceptible to nighttime dim light than male mice (Chen et al., 2021). Light information includes light intensity, duration, and wavelength. Our previous studies demonstrated the influence of light wavelength factors on the hippocampal inflammatory response in mice. Under dWL and dBL conditions, the density of CA3 and CA1 neurons in the mice hippocampus significantly decreased, while the number and activation of microglia in the hippocampus significantly increased, leading to oxidative stress and neuroinflammation (Liu et al., 2022). Furthermore, several studies have indicated that Dim Light at Night changes the inflammatory response in mice, particularly by boosting the expression of pro-inflammatory cytokines in microglia (Fonken et al., 2013a; Fonken et al., 2013b). Given the exacerbating effect of dim light on neuroinflammation at night, it is critical to find a solution. Melatonin, which is secreted by the pineal gland, has anti-inflammatory, antioxidant, and antidepressant effects, making it thought to offer significant therapeutic potential for neurodegenerative illnesses (Xu et al., 2020). Melatonin is an essential regulator of neuroinflammation. Tahir Ali et al. found that melatonin reduces LPS-induced activation of astrocytes and microglia while improving neuroinflammation (Ali et al., 2020). Our study discovered that melatonin can reduce the level of hippocampal inflammation caused by dBL, diminish the expression of CD80 in microglia, and block the activation of microglia.

Corticosterone is a hormone that is secreted rhythmically and is easily influenced by light. The Dim Light at Night can affect corticosterone secretion, and this effect is related to the species and the lighting conditions used, such as light wavelength, light intensity, etc (Rumanova et al., 2020). In previous studies, plasma corticosterone levels in mice were significantly increased under constant light at night with white light (dWL) and constant light at night with blue light (dBL). Corticosterone was found to activate BV2 cells, leading to the expression of pro-inflammatory cytokines and proteins (Liu et al., 2022). Recent studies have confirmed that corticosterone is involved in stress-induced neuroinflammatory responses, thus playing a significant role in the pathogenesis of diseases like depression and post-traumatic stress disorder. For example, Bin Xu et al. demonstrated that corticosterone can activate the MAPK signaling pathway in microglia in the dentate gyrus (DG) region of the hippocampus in mice, promoting the pro-inflammatory activation of microglia (Xu et al., 2019). Nod-like receptor protein 3 (NLRP3) inflammasome is expressed in microglia and plays a crucial role in the release of proinflammatory factors from microglia. It is able to activate caspase-1 to produce inflammatory factors such as IL-1β (Dong et al., 2020). Feng et al. found that glucocorticoids can increase NF-kB nuclear transcription in hippocampal microglia, which then activates the NLRP3 inflammasome, disrupting the homeostasis of hippocampal microglia (Feng et al., 2019). Therefore, *in vitro* experiments, we used corticosterone to stimulate BV2 cells to mimic the effects of dark blue light at night on hippocampal microglia. The results showed that the expressions of CD80, pp65, and NLRP3 in BV2 cells significantly increased after corticosterone treatment. This indicates that corticosterone effectively stimulated the activation of microglia by activating the NLRP3 inflammasome through NF-kB signaling. This indicates that our method of modeling BV2 cells with corticosterone stimulation *in vitro* is feasible. It has been reported that microglial activation induces neuronal cell death through the overexpression of pro-inflammatory mediators, leading to neuronal disorder and death (Jebelli et al., 2014). Melatonin regulates the state of microglia to protect hippocampal neurons, a finding that was also confirmed in our study. We found that treating the BV2 cell culture medium with melatonin significantly increased the cell viability of HT22 cells. Unfortunately, we did not detect the expression of inflammatory factors in the BV2 cell culture.

The regulatory effect of melatonin on NF-kB and NLRP3 in BV2 cells has been confirmed in several studies. For example, melatonin preconditioning attenuates manganese and LPS-induced NF-kB activation and reduces the expression of iNOS and nitric oxide (NO) in BV2 cells (Park and Chun, 2017). Tang et al. showed that melatonin therapy improved the activation of the NLRP3 inflammasome in thrombine-induced activated BV2 cells, and that this improvement was associated with a reduction in ROS production (Tang et al., 2020). We investigated the impact of melatonin on corticosterone-induced microglia activation and observed that the inclusion of melatonin in BV2 cells significantly decreased corticosterone-induced inflammation. This reduction was evident in the decreased expression of inflammatory proteins PP65, NLRP3, and the M1 marker CD80 in microglia. This suggests that melatonin may reduce NF-kB transcription and control the activation of the NLRP3 inflammasome to improve the overactivation of microglia by corticosterone. In addition, Zheng Ran et al. reported that melatonin can reduce MPTP-induced microglial activation, inhibit the activity of the NLRP3 inflammasome, and suppress IL-1β secretion (Zheng et al., 2021). Melatonin treatment significantly inhibited sleep deprivation (SD)-induced hippocampal NF-κB activation and the production of pro-inflammatory cytokines, demonstrating anti-inflammatory effects (Wang et al., 2021). These studies suggest that melatonin is involved in the activation of microglia NF-kB signaling pathway and NLRP3, influencing microglia polarization to control brain inflammation.

At present, there are few reports on how melatonin regulates microglia. Melatonin is a pleiotropic molecule that exerts various physiological effects primarily through receptor-dependent signaling cascades. The main membrane receptors for melatonin are MT1 and MT2, which are G-protein-coupled receptors. Gao Yuan et al. indicated that melatonin alleviates inflammation-mediated brain edema and neurological deficits by activating the MT2/IL-33/*F*th pathway (Gao et al., 2023). In another study, pretreatment with 100 nM melatonin prevented the overexpression of TNF-α induced by methamphetamine exposure. Conversely, the anti-inflammatory effects of melatonin were eliminated when MT2 was knocked down using siRNA (Wongprayoon and Govitrapong, 2015). Our results showed that melatonin could significantly improve the secretion of corticosterone-induced inflammatory factors in BV2 cells. However, the beneficial effect of melatonin was no longer observed after the addition of the MT2 receptor blocker 4-P-PDOT. This suggests that melatonin alleviated corticosterone-induced activation of BV2 cells primarily through MT2 receptors. A study has also shown that the effects of melatonin on microglia are primarily receptor-mediated and partly dependent on SIRT1 activation (Merlo et al., 2023). It can be seen that the receptor action pathway of melatonin is crucial for regulating microglia polarization.

In summary, this study showed that melatonin alleviates nocturnal blue-induced hippocampal neuroinflammation by modifying phenotypic markers of microglia, thereby reducing neuronal damage. Melatonin regulates microglial activation towards the M1 proinflammatory type through the NF-kB - NLRP3 pathway, and MT2 plays a crucial role in the modulation of microglial polarization. This finding reflects the beneficial role of Mel-MT2 in regulating microglia polarization, offering new insights into the management of nerve disorders triggered by light pollution, such as dim light at night. However, there are also limitations in this study. First, we only analyzed the role of melatonin from the perspective of the receptor pathway. Whether the non-receptor pathway of melatonin also has the ability to regulate microglia polarization remains to be clarified. Second, this study only investigated the effects of melatonin on microglia in the hippocampus. Studies have shown that light not only affects the activation of microglia in the hippocampus but also has an effect on microglia in the basolateral amygdala (BLA), anterior cingulate cortex (ACC), and other brain regions (Costello A et al., 2023). Therefore, whether melatonin can improve neuroinflammation in these brain regions remains to be studied. Third, this experiment uses rodents as test models, and it is important to consider species differences when translating *in vivo* and *in vitro* test results to humans.

## Data Availability

The raw data supporting the conclusion of this article will be made available by the authors, without undue reservation.
